# Genetic Study of Cerebral Small Vessel Disease in Chinese Han Population

**DOI:** 10.3389/fneur.2022.829438

**Published:** 2022-03-25

**Authors:** Yunchao Wang, Changhe Shi, Yusheng Li, Wenkai Yu, Sen Wei, Yu Fan, Chengyuan Mao, Zhihua Yang, Lulu Yu, Zichen Zhao, Shanshan Li, Yuan Gao, Yuming Xu

**Affiliations:** Department of Neurology, The First Affiliated Hospital of Zhengzhou University, Zhengzhou University, Zhengzhou, China

**Keywords:** mutations, monogenic, cerebral small vessel disease, Chinese Han population, genetic study

## Abstract

Cerebral small vessel disease (CSVD) is a syndrome of clinical, neuroimaging, and neuropathological manifestations caused by disorders that affect small cerebral vessels. Although the pathogenesis of the disease remains unclear, some studies have demonstrated that genetic variants contribute to the development of CSVD. Our study aimed to explore the genetic characteristics of CSVD in the Chinese Han population. We enrolled 182 sporadic CSVD Chinese Han patients whose magnetic resonance imaging results showed grade 2-3 white matter lesions. Target region sequencing of seven monogenic CSVD-related genes, including *NOTCH3, HTRA1, COL4A1, COL4A2, GLA, TREX1*, and *CTSA*, was performed, and we identified pathogenic variants by screening the sequencing results and functional predictive analysis. All variants were predicted to be pathogenic by the SIFT Score, Polymorphism Phenotyping-2 score, Mutation Taster, Splice site score calculation, and MaxEntScan. All variants were validated in 300 healthy controls. In total, eight variants were identified in patients with CSVD, including five novel variants, c.1774C>T (*NOTCH3*), c.3784C>T (*NOTCH3*), c. 1207C>T (*HTRA1*), and c. 1274+1G> A (*HTRA1*), c.1937G>C (*COL4A1*) and three reported mutations. None of these variants were present in 300 healthy controls. No pathogenic variants in *COL4A2, GLA, TREX1*, and *CTSA* were detected. This study identified five novel variants in CSVD-related genes in Chinese Han patients with sporadic CSVD. Our results expand the genetic profile of CSVD.

## Introduction

Cerebral small vessel disease (CSVD) is a syndrome of clinical, neuroimaging, and neuropathological manifestations caused by disorders that affect small cerebral vessels, including arteries, arterioles, capillaries and venules, in the brain (1). In the United States and Europe, CSVD accounts for 15–26% of cases of ischemic stroke (2–5), and the proportion ranges from 25 to 54% in Asia (6–10). Several studies have suggested that ethnic differences, vascular risk factors, genetic factors, and environmental sensitivities play an essential role in the mechanism and etiology of CSVD (11), with genetic factors being shown to have a significant effect on CSVD.

Most CSVD is sporadic (12), and Mendelian forms only account for 5% of CSVD patients (13). Monogenic CSVD has been proven to be caused by specific genetic mutations, including cerebral autosomal dominant arteriopathy with subcortical infarcts and leukoencephalopathy (CADASIL) (14, 15), cerebral autosomal recessive arteriopathy with subcortical infarcts and leukoencephalopathy (CARASIL) (15), *COL4A1/2*-related CSVD (16–18), Fabry's disease (19), retinal vasculopathy with cerebral leukodystrophy (RVCL) (14), and cathepsin A–related arteriopathy with strokes and leukoencephalopathy (CARASAL) (20). Overall, understanding genetic factors involved in CSVD will be helpful for improving the diagnosis and treatment of these monogenic diseases as well as sporadic CSVD.

In this study, we sequenced common monogenic CSVD-related genes, including *NOTCH3, HTRA1, COL4A1/2, GLA, TREX1*, and *CTSA*, in 182 Chinese Han patients with CSVD and 300 healthy subjects to investigate the characteristics of CSVD in the Chinese Han population.

## Materials and Methods

### Enrollment

We enrolled 182 unrelated patients with CSVD who were admitted to the Department of Neurology, First Affiliated Hospital of Zhengzhou University, from March 2017 to July 2018. The diagnosis of CSVD was performed by neurologists and neuroimaging (11). The inclusion criteria were as follows: (1) age over 18 years; (2) marked leukoencephalopathy defined as Fazekas grade 2 or grade 3 on neuroimaging (21). The exclusion criteria were as follows: (1) acute cerebral infarction lesions (the lesion diameter of diffusion-weighted imaging was more than 20 mm); (2) Acute cerebral hemorrhage, acute subarachnoid hemorrhage, history of cerebral vascular malformation or subarachnoid hemorrhage caused by cerebral aneurysm, or untreated cerebral aneurysm (diameter > 3 mm); (3) monogenic CSVD with known mutations; (4) dementia affected by confirmed neurodegenerative diseases, such as Alzheimer's disease (AD), Parkinson's Disease; (5) suffering from evident white matter lesions of non-vascular origin, such as multiple sclerosis, adult white matter dysplasia, and metabolic encephalopathy; (6) mental diseases diagnosed according to the Diagnostic and Statistical Manual of Mental Disorders V (DSM-V), intracranial infection, traumatic brain injury, or brain tumors. We recruited 300 healthy controls. This study was approved by the Ethics Committee of the First Affiliated Hospital of Zhengzhou University.

### Magnetic Resonance Imaging

All patients with CSVD underwent imaging examination at the First Affiliated Hospital of Zhengzhou University. Demographic information, clinical symptoms, and imaging findings were recorded for all patients with CSVD. Neuroimaging was assessed independently by two trained neurologists. The severity of white matter hyperintensity was quantified on T2-weighted images or fluid-attenuated inversion recovery sequences of brain MRI; cerebral microbleeds were analyzed on T2^*^-weighted gradient-recalled echo or susceptibility-weighted sequences (11). The Fazekas scale was used to assess the severity of white matter hyperintensity.

### DNA Extraction and Next-Generation Sequencing

Genomic DNA was isolated from 2 ml peripheral blood collected following the manufacturer's standard procedure. For each of the seven genes examined, both coding and non-coding (regulatory) regions were included in the sequencing target. The regulatory genomic regions comprised the promoter region (defined as 2 kb upstream of the transcription start site), 5′ untranslated region (5′ UTR), and intron-exon boundaries (50 bp). After sequencing primer hybridization, base incorporation was carried out using a HiSeq 2000 (Illumina, Inc., San Diego, CA) following the manufacturer's standard sequencing protocols for 101 cycles of sequencing per read to generate paired-end reads, including 100 bp at each end and six bp of the index tag.

### Bioinformatics Analysis

Raw sequencing data were demultiplexed into individual Fastq read files with Illumina's bcl2fastq v2.16.0.10 based on unique index pairs. Low-quality (*Q* < 15) reads/bases were trimmed using the fastx tool. High-quality reads were aligned to the NCBI human reference genome (hg19) using Burrows Wheeler Aligner (BWA) software, which can build assemblies by mapping short reads to a reference genome using default parameters (22). SNV (single-nucleotide variant) calling was performed using both GATK (23) and Varscan (24), and the called SNV data were then combined. The SNV data in each sample were first compared to all variations annotated in dbSNP147 along with data from the 1000 Genomes Project. After this analysis, all newly identified variations were fully annotated. All filtering and annotation were performed using ANNOVAR (25). Variants that had a depth of coverage <10 were removed. The functional effect of non-synonymous SNVs was assessed by the SIFT score (http://sift.jcvi.org/www/SIFT), PolyPhen-2 (http://genetics.bwh.harvard.edu/pph2/) and Mutation Taster (http://www.mutationtaster.org) (26–28). Splice site mutations were analyzed using the bioinformatics tools SSS (http://rulai.cshl.edu/new_alt_exon_db2/HTML/score.html) and MaxEntScan (http://hollywood.mit.edu/burgelab/maxent/Xmaxentscan_scoreseq_acc.html). Finally, we analyzed the variants according to the American College of Medical Genetics (ACMG) practice guidelines.

Any SNV recorded in dbSNP147 with a minor allele frequency of ≥1% in the 1000 Genomes Project database, ≥1% in our dataset, and with missing calls in <10% of subjects was considered a single-nucleotide polymorphism and included in subsequent individual-variant association analysis; SNPs failing the Hardy-Weinberg equilibrium test at a significance level of 0.0001 were removed. SNVs in genes not reported in dbSNP and with a Global minor allele frequency (MAF) <0.01 in the 1000 Genomes Project database (http://www.1000genomes.org/home), the ExAC03 dataset (Exome Aggregation Consortium) (https://www.ncbi.nlm.nih.gov/bioproject/PRJEB8661), the esp6500 dataset (NHLBI GO Exome Sequencing Project Frequency of 6500 samples of full exon sequencing) (http://evs.gs.washington.edu/EVS/) and the GnomAD dataset (http://gnomad.broadinstitute.org) were considered to be rare variants and were included for subsequent gene-level burden analysis.

### Validation by Sanger Sequencing

Sanger sequencing was performed to confirm the remaining eight candidate variants among the seven samples identified by NGS. Specific primers for the corresponding exons of *NOTCH3, HTRA1, and COL4A1* were designed using Primer 6 Software. All these exons and exon-intron boundaries were amplified from genomic DNA by polymerase chain reaction (PCR). The purified PCR products were genotyped by Sanger sequencing. DNASTAR Lasergene MegAlign (v7.1.0) and Chromas (v2.33) were used to conduct sequence alignment. The primers used for all variants are shown in Supplementary Table 1.

## Results

### Variant Filtering

**Figure 1:**
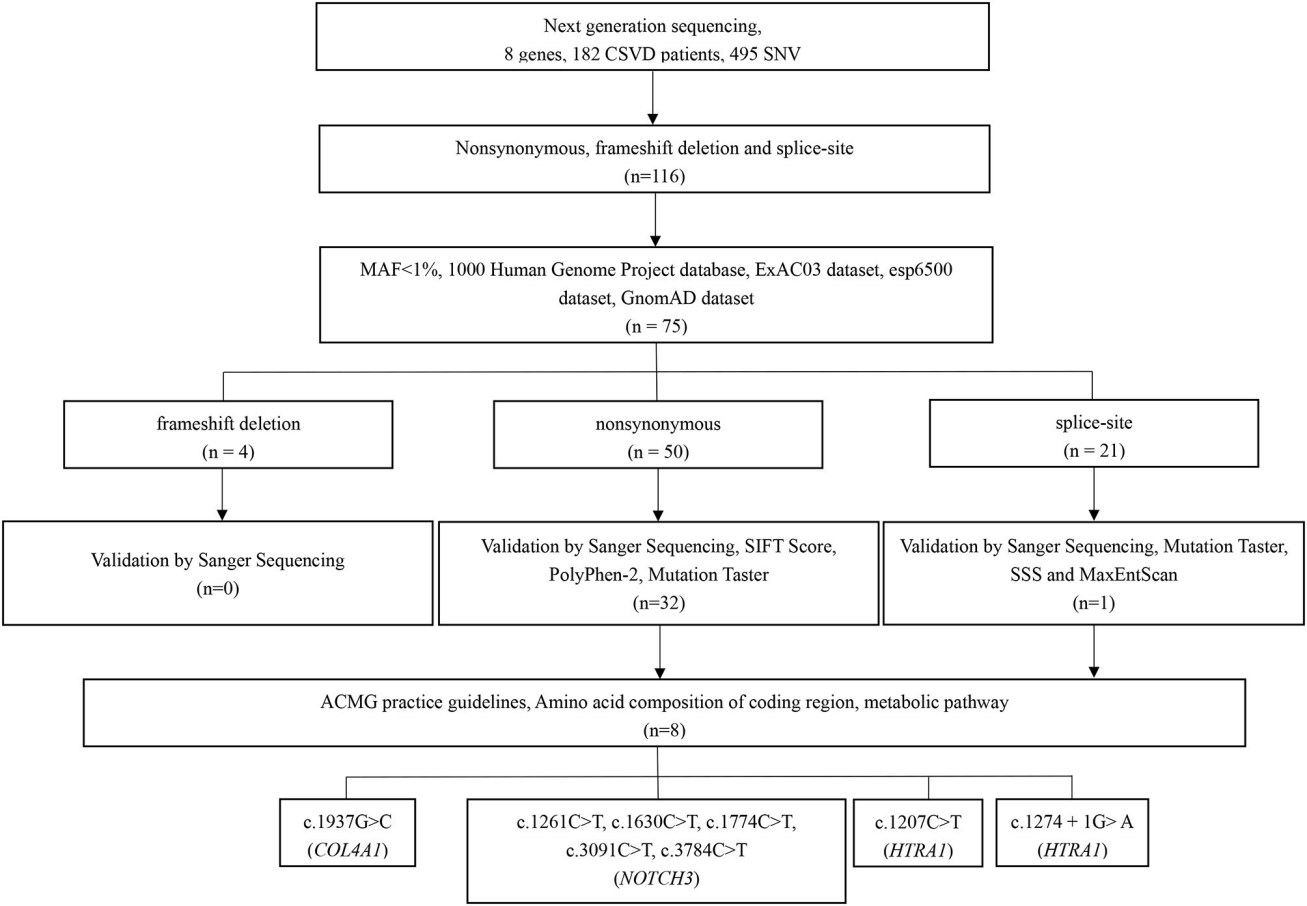
Filtration process and the remaining variants.

In total, 495 SNVs (single-nucleotide variants) were identified initially on the basis of NGS, with a mean target depth of 116 ×. For the first step, 379 variants located in introns (except for splice site variants) and synonymous variants were excluded. Considering the specific ethnicity being studied, the remaining 116 variants were then filtered based on the 1000 Genomes Project database, the ExAC03 dataset, the esp6500 dataset, and the GnomAD dataset to exclude those with minor allele frequencies >1% (41 variants). Among the 75 remaining variants, four were frameshift variants, 50 synonymous variants, and 21 splice-site variants. All four frameshift variants were excluded through validation by Sanger sequencing. Among the 50 synonymous variants, 18 were excluded due to their benign *in silico* prediction based on validation by Sanger sequencing, the SIFT score, the PolyPhen-2 score, and Mutation Taster. For the 21 splice site variants, 20 were excluded according to validation by Sanger sequencing, Mutation Taster, SSS, and MaxEntScan. Based on the amino acid composition of the coding region and metabolic pathway, eight variants in 3 genes were ultimately retained after the strict filtering process (Figure 1). The variants considered pathogenic are missense or splice site variants at highly conserved sites. The amino acids influenced by these seven missense variants are highly evolutionally conserved among the different species (Supplementary Figure 1).

**Figure 2:**
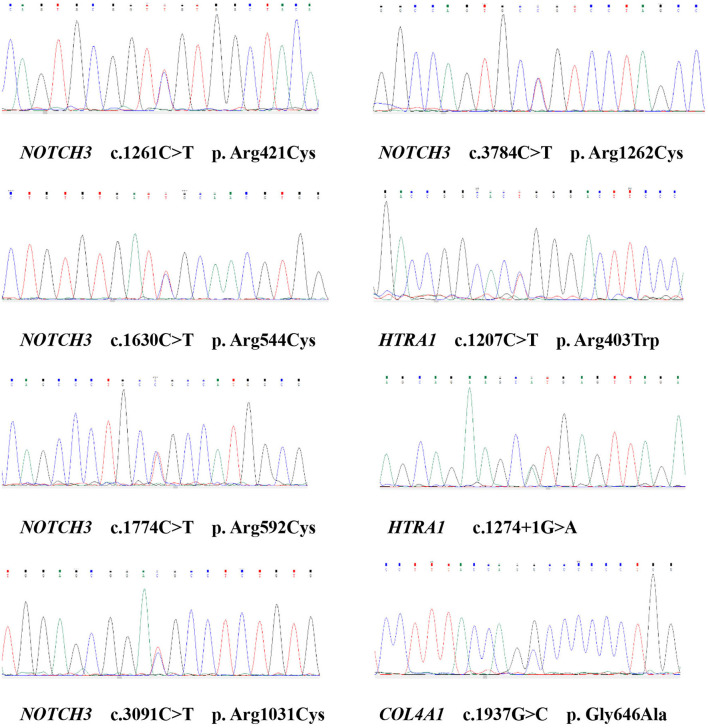
Sequencing chromatograms showed the variants of the *NOTCH3, HTRA1, COL4A1*.

We identified eight variants in patients with CSVD, including c. 1261C>T (*NOTCH3*), c.1630C>T (*NOTCH3*), c.1774C>T (*NOTCH3*), c.3091C>T (*NOTCH3*), c.3784C>T (*NOTCH3*), c. 1207C>T (*HTRA1*), c. 1274 + 1G> A (*HTRA1*), and c.1937G>C (*COL4A1*). One patient carried c.3091C>T (*NOTCH3*) and c.1937G>C (*COL4A1*). The remaining six patients each carried one variant. Except for c. 1274 + 1G> A (*HTRA1*), the seven missense variants identified in this study involve highly evolutionarily conserved residues. No pathogenic variants in *COL4A2, GLA, TREX1*, and *CTSA* were detected. Moreover, the variants were absent in the 300 unrelated ethnically matched healthy controls. The results are presented in Table 1 and Figure 2. A comparison of clinical features between patients with pathogenic variants and those without pathogenic variants was presented in Supplementary Table 2.

**Table 1:** Eight pathogenic variants identified in CSVD-related genes in 7 SVD patients.

**Gene and location**					**Bioinformatics prediction**				**Population**				
**Gene**	**Exon**	**SNP ID**	**Nucleotide**	**AminoAcid**	**ACMG^a^**	**SIFT^b^**	**POLYPhen-2^c^**	**MutationTaster^d^**	**1,000 g**	**ExAC03**	**esp6500**	**GnomAD**	**300 control**
*NOTCH3*	8	NA	c. 1261C>T	p. Arg421Cys	US	D	D	D	NF	NF	NF	NF	NF
*NOTCH3*	11	rs201118034	c. 1630C>T	p. Arg544Cys	US	T	P	D	0.0002	0.0003	NF	0.0003	NF
*NOTCH3*	11	rs764148985	c. 1774C>T	p. Arg592Cys	US	D	D	D	NF	NF	NF	0.0001	NF
*NOTCH3*	19	NA	c. 3091C>T	p. Arg1031Cys	US	T	D	D	NF	NF	NF	NF	NF
*NOTCH3*	23	NA	c. 3784C>T	p. Arg1262Cys	US	D	P	D	NF	NF	NF	NF	NF
*HTRA1*	8	rs147459330	c. 1207C>T	p. Arg403Trp	US	D	D	D	0.001398	0.0005	NF	8.20E-06	NF
*HTRA1*	8	rs751805574	c. 1274+1G>A	NA	P	NA	NA	D	NF	8.24E-06	NF	4.06E-06	NF
*COL4A1*	27	rs532972509	c.1937G>C	p. Gly646Ala	US	D	D	D	0.0002	8.84E-06	7.7E-05	0.0004	NF

*NA, Not Available; NF, Not Found.*

*The amino acid is based on the corresponding cDNA position according to the reference sequences of NOTCH3 (GRCh38, NM_000435), HTRA1(GRCh38, NM_002775), COL4A1(GRCh38, NM_001845).*

a *P, Pathogenic; US, Uncertain significance.*

b *D, Damaging; T, Tolerated.*

c *P, Possibly Damaging; D, Probably Damaging.*

d *D, Disease Causing*.

### NOTCH3

We found 22 non-synonymous and 16 synonymous variants. Among the 22 non-synonymous variants, the SIFT score, the PolyPhen-2 score, and the functional predictions of Mutation Taster indicated that five are pathogenic: c. 1261C>T (*NOTCH3*), c.1630C>T (*NOTCH3*), c.1774C>T (*NOTCH3*), c.3091C>T (*NOTCH3*), c.3784C>T (*NOTCH3*). These variants may cause the epidermal growth factor repeat (EGFR) to acquire a cysteine, causing a change in the abundance of cysteine residues. The frequencies of these five variants are low in the 1000 Genomes Project database, the ExAC03 dataset, the esp6500 dataset, and the GnomAD dataset (<1‰). Finally, we identified five *NOTCH3* heterozygous variants, each of which was carried by one patient. In this study, the frequency of CSVD patients carrying the pathogenic *NOTCH3* variants was 2.75% (Table 1).

These five patients exhibited varying degrees of cognitive impairment. One patient had hypertension and stroke, two patients had hypertension, stroke, and a family history of stroke. Two patients did not have any risk factors for cerebrovascular disease (Table 2).

**Table 2:** The clinical characteristics of the 7 SVD patients with pathogenic variants.

**Patients**	**Gene**	**Variants**	**Sex**	**Age of onset**	**PWMH**	**DWMH**	**LH**	**CMB**	**Stroke**	**Risk factors**	**MMSE**	**MoCa**
1	*NOTCH3*	c. 1261C>T	M	51	3	3	+	–	CI	HTN	14	7
	*COL4A1*	c. 1937G>C										
2	*NOTCH3*	c. 1630C>T	F	63	3	3	–	+	–	–	10	4
3	*NOTCH3*	c. 1774C>T	M	64	2	1	+	+	CI	HTN	16	5
4	*NOTCH3*	c. 3091C>T	F	46	3	3	+	–	–	–	28	26
5	*NOTCH3*	c. 3784C>T	M	76	3	3	+	NA	CI	HTN	NA	NA
6	*HTRA1*	c. 1207C>T	F	56	2	3	+	+	CI	HTN, DM, CAD	19	7
7	*HTRA1*	c. 1274+1G>A	M	66	3	2	+	NA	CI, ICH	HTN, DM, HHcy	NA	NA

*M, man; F, female; CI, cerebral infarction; PWMH, Periventricular White matter hyperintensity; DWMH, Deep White matter hyperintensity; LI, Lacunar Infarcts; CMB, Cerebral Microbleeds; ICH, intracranial hemorrhage; HTN, hypertension; DM, diabetes mellitus; HHcy, hyperhomocysteinemia; CAD, coronary artery disease; MMSE, Mini-Mental State Examination; MoCa, Montreal Cognitive Assessment; NA, Not Available; + indicates present; – indicates absent*.

### HTRA1

We found three non-synonymous variants, six synonymous variants, one insertion frameshift variant, and four splice site variants. Among the three non-synonymous variants located in exons, the SIFT score, the PolyPhen-2 score, and the functional prediction of Mutation Taster predicted only p.R403W (*HTRA1*) to be pathogenic. The frequency of c. 1207C>T (*HTRA1*) in the 1000 Genomes Project database is 1.39‰, and the frequency of the gene in the ExAC03, esp6500, and GnomAD gene databases is very low (<1‰). Among the four splice site variants, SSS and MaxEntScan found that only c. 1274+1G> A (*HTRA1*) might affect the normal splicing of mRNA. As a result, the physiological function of serine protease one would be disrupted. c. 1274+1G> A (*HTRA1*) has a very low gene frequency (<1‰) in the ExAC03, esp6500, and GnomAD gene databases, indicating a rare variant. At the same time, Mutation Taster functional prediction showed that c. 1274+1G> A (*HTRA1*) is pathogenic. Finally, we identified two rare *HTRA1* heterozygous variants; the relationship between these two variants and CSVD has never been reported, and each variant was carried by one patient. In this study, the frequency of CSVD patients carrying the pathogenic *HTRA1* variants was 1.10% (Table 1).

Two patients displayed varying degrees of cognitive dysfunction. Both had hypertension and diabetes mellitus (DM). One patient had hyperhomocysteinemia, ischemic and hemorrhagic strokes, and the other had coronary artery disease (CAD). However, none of the patients had a family history of similar diseases (Table 2).

### COL4A1

We found 11 non-synonymous variants and 18 synonymous variants. Only one synonymous variant would cause a change from glycine to alanine. SIFT score, PolyPhen-2 score, and Mutation Taster functional prediction showed that c.1937G>C (*COL4A1*) is pathogenic; the frequency of the variant is relatively low in the 1000 Genomes Project database, the ExAC03 dataset, the esp6500 dataset, and the GnomAD dataset (<1‰), indicating a rare variant. The frequency of the remaining non-synonymous variants is more than 1% in the 1000 Genomes Project database, and we believe that these variants are not likely to cause disease. Finally, we identified a rare heterozygous variant that has never been reported, which was carried by one patient. In this study, the frequency of CSVD patients carrying the pathogenic *COL4A1* gene variants was 0.55% (Table 1).

This patient exhibited severe cognitive dysfunction, hypertension, recurrent ischemic stroke, and a severe family history of stroke. this patient also carried a pathogenic *NOTCH3* variant (Table 2).

## Discussion

Eight heterozygous variants were identified in this study, including c. 1261C>T (*NOTCH3*), c. 1630C>T (*NOTCH3*), c. 1774C>T (*NOTCH3*), c. 3091C>T (*NOTCH3*), c. 3784C>T (*NOTCH3*), c. 1207C>T (*HTRA1*), c. 1274 + 1G> A (*HTRA1*), and c. 1937G>C (*COL4A1*). Among them, c. 3091C>T (*NOTCH3*) and c. 1937G>C (*COL4A1*) were carried by the same patient; for the remaining six variants, each was carried by one patient. We did not detect pathogenic variants in *COL4A2, GLA, TREX1*, or *CTSA*, and no pathogenic variants were found in 300 healthy controls.

CADASIL is one of the most common single-gene disorders of cerebral small blood vessels caused by mutations in the *NOTCH3* gene on chromosome 19q12 (29, 30). At present, there are more than 500 CADASIL families reported in the world, and more than 230 disease-causing mutations have been detected in the *NOTCH3* gene (31, 32). The mutations are mainly concentrated in the exon 2-24 region (33–35). In this study, five heterozygous variants were found in exon 8, exon 11, exon 19, and exon 23. Opherk et al. reported the mutation c. 1261C>T (*NOTCH3*) in a retrospective study of 411 CADASIL patients in 2004 (32, 36), and Oberstein et al. (37) described the mutation c.1630C>T (*NOTCH3*) based on direct sequence analysis of an abnormal *NOTCH3* gene in patients from 11 families in 1999. Joutel et al. (33) reported the c. 3091C>T (*NOTCH3*) mutation in 50 unrelated white patients with a strong suspicion of CADASIL in 1997. c. 1774C>T (*NOTCH3*) and c. 3784C>T (*NOTCH3*) have not been reported before. The five heterozygous variants are distributed in the EGFR domains of the *NOTCH3* gene and are associated with CADASIL. These variants cause the EGFR domain to acquire another cysteine, resulting in a change in the number of this amino acid (29, 30, 38). According to electron microscopy (EM), the pathognomonic feature of CADASIL is the accumulation of granular osmiophilic material (GOM) in indentations of vascular smooth muscle cells (VSMCs) or in the extracellular space in close vicinity to VSMCs, leading to changes in cerebral vascular structure and function, such as leukoaraiosis, ischemic cerebrovascular disease, and cognitive impairment (1, 39). We also found some variants that did not affect cysteines, but the results of functional prediction suggest that the variants may cause disease. Previously, it has been reported that some variants detected in CADASIL patients do not cause changes in cysteines (40, 41). Moreover, it is interesting that some common *NOTCH3* gene SNPs can cause CADASIL and increase the risk of age-related WMH in patients with hypertension (42). Therefore, in some cases, CADASIL appears to be derived from atypical *NOTCH3* mutation that does not affect cysteine. The pathogenicity of these variants is currently controversial (15, 43, 44) and needs further research.

In addition, CARASIL is a very rare autosomal recessive CSVD caused by biallelic mutations of the *HTRA1* gene (high-temperature requirement protease A1) (45). In this study, we found two variants, p. R403W and c. 1274+1G>A, that completely or partially abolish *HTRA1* protease activity. The missense variant is located in the C-terminal PDZ domain, which participates in central regulation. As the serine protease 1 (*HTRA1*) chaperone (PDZ domain tightly closed) and proteolysis (PDZ domain open) functions are regulated by the PDZ domain, the variant affects these activities of the protein encoded by *HTRA1*. c. 1274+1G > A is located in the intron near exon 8. The variant affects the initial nucleotide of the splice donor site, impacting the normal splicing of mRNA and impairing the physiological function of *HTRA1*. To date, 44 mutations have been reported in this gene, most of which are missense and heterozygous variants (46). Although the exact mechanism responsible for a single heterozygous *HTRA1* mutation causing CSVD is still not fully clear, impaired *HTRA1* protease function seems to be a core feature of *HTRA1*-related autosomal dominant CSVD, and relevant *in vitro* functional assays may help to evaluate the pathogenicity of *HTRA1* variants.

The *COL4A1* and *COL4A2* genes are located on chromosome 13q34 and encode collagen type IV alpha 1 (COL4A1) and collagen type IV alpha 2 (COL4A2), respectively (47). Type IV collagen is the main component of all basement membranes in humans. Mutations in the *COL4A1* gene contribute to a broad spectrum of disorders involving a specific phenotype comprising hereditary angiopathy, nephropathy, aneurysms, and cramps (HANAC) (47). Dominant missense mutations in the *COL4A1* gene result in a rare familial stroke characterized by deep ICH, lacunar ischemic stroke, and WMH (48, 49). More than 30 types of mutations have been reported in the *COL4A1* gene thus far, and the number is rapidly increasing (18). COL4A1 and COL4A2 assemble to form a heterotrimeric helix with a constant 2:1 ratio [proα1(IV)] 2 [proα2 (IV)]. The triple-helical domain consists of a triple amino acid repeat sequence, Gly-Xaa-Yaa, where Xaa and Yaa can be any residue. This sequence is interrupted by several motifs containing cysteine residues, which are essential in providing flexibility to the collagen IV network and possible binding sites for intermolecular cross-linking (18, 50, 51). In this study, we found a new variant, namely, c.1937G>C. This variant causes glycine to become an alanine, leading to a change in the structure of the heterotrimeric helix in the basement membrane. It destroys the structure and stability of the vascular basement membrane, causing destruction of the vessel wall structure and a series of vascular diseases. Although *COL4A1* gene mutations are known to lead to instability and defects in the basement membrane, the exact mechanism linking mutations and phenotypes is presently unknown.

## Conclusion

Our study aimed to explore the genetic characteristics of the sporadic CSVD in the Chinese Han population. The results expand the genetic profile of CSVD. We explored the status of multiple known monogenic CSVD genes in the sporadic population. The results of our study suggest that variants in these genes may be associated with CSVD in the sporadic population, providing substantial evidence for individuals susceptible to sporadic CSVD. This study also reminds clinicians to pay attention to the screening of genetic factors in sporadic patients with CSVD. This will help improve the diagnosis and treatment of sporadic CSVD. However, due to the small number of patients included in this study and they all came from the same hospital, there was a certain degree of bias. The pathogenic mechanisms involved need to be further studied in cell and animal models to provide a basis for further research and treatment.

## Data Availability Statement

The original contributions presented in the study are included in the article/Supplementary Material, further inquiries can be directed to the corresponding authors.

## Ethics Statement

The studies involving human participants were reviewed and approved by Ethics Committee of First Affiliated Hospital of Zhengzhou University. The patients/participants provided their written informed consent to participate in this study. Written informed consent was obtained from the individual(s) for the publication of any potentially identifiable images or data included in this article.

## Author Contributions

YW, YX, and YG contributed to the conception and design of the study. WY, LY, ZZ, and SL contributed to the acquisition. YW drafted the text and prepared the figures. YW, YX, YG, CS, YL, WY, SW, YF, CM, and ZY contributed to the analysis of data. All authors critically reviewed the manuscript and approved it as submitted.

## Funding

This work was supported by Henan Science and Technology Innovation Talent Project in 2015 to YX (Grant Number 154200510017), National Key Research and Development Program of China to YG (Grant Number 2018YFC1311303), the National Natural Science Foundation of China to YX (Grant Numbers U1904207, 81530037, and 91849115), and the Non-profit Central Research Institute Fund of Chinese Academy of Medical Sciences (Grant Number 2020-PT310-01).

## Conflict of Interest

The authors declare that the research was conducted in the absence of any commercial or financial relationships that could be construed as a potential conflict of interest.

## Publisher's Note

All claims expressed in this article are solely those of the authors and do not necessarily represent those of their affiliated organizations, or those of the publisher, the editors and the reviewers. Any product that may be evaluated in this article, or claim that may be made by its manufacturer, is not guaranteed or endorsed by the publisher.
