# Supplementary material for: Genetic Study of Cerebral Small Vessel Disease in Chinese Han Population
Source: Front Neurol. 2022 Mar 25;13:829438. doi: 10.3389/fneur.2022.829438 (PMC8990910; doi:10.3389/fneur.2022.829438)
Supplement: Supplementary file 1 [file Data_Sheet_1.PDF]

Supplementary Material

Supplementary Table 1. Primer sequences used for sanger sequencing.

Gene	Exon	Nucleotide	Primer sequences
NOTCH3	8	c.C1261T	F: 5’-ACGCCCACACCGATCGCACT-3’<br>R: 5’-CCATCCGCGGGCTTCTCTGT-3’
NOTCH3	11	c.C1630T	F: 5’-CCTAGACCTGGTGGACAA-3’<br>R: 5’-CACAGTCGTCAATGTTCAC-3’
NOTCH3	11	c.C1774T	F: 5’-GCGGAGCCTGACCCTCTTGG-3’<br>R: 5’-CTCCAGGTGTGCTGTTTCTGC-3’
NOTCH3	19	c.C3091T	F: 5’-AGTTTCTCCCAGACTACCC-3’<br>R: 5’-CCATTCGGCTCACACTAG-3’
NOTCH3	23	c.C3784T	F: 5’-AGGTAAGCGTTGGCGAAG-3’<br>R: 5’-ATTTTTAGTAGAGACGGGGTTT-3’
HTRA1	8	c.C1207T	F: 5’-ACTCTGGTAGACAGGCAAT-3’<br>R: 5’-ACTCCAACTCACGCTTCT-3’
HTRA1	8	c.1274+1G>A	F: 5’-ACTCTGGTAGACAGGCAAT-3’<br>R: 5’-ACTCCAACTCACGCTTCT-3’
COL4A1	27	c.G1937C	F: 5’-GTGTGGTCCTCATTCCTTC-3’<br>R: 5’-GAAACTCTCGTGGTATCCC-3’

Supplementary Table 2. Comparison of demographic and clinical characteristics between the cerebral small vessel disease patients with pathogenic variants and without pathogenic variants.

Variables	Patients with pathogenic mutations (n=7)	Patients without pathogenic mutations (n=175)	p value
**Age of onset (year)**	60.29 ± 10.08	65.51 ± 9.37	0.151
**Sex (male)**	4 (57.1%)	110(62.9%)	1.000
**Clinical symptoms**			
Muscle weakness	3 (42.9%)	80(45.7%)	1.000
Speech impairment	2 (28.6%)	18 (10.3%)	0.172
Dizzy	0 (0.0%)	41 (23.4%)	0.352
Sensory disturbance	1 (14.3%)	88 (50.3%)	0.118
Headache	1 (14.3%)	8 (4.6%)	0.303
Migraine	1 (14.3%)	3 (1.7%)	0.146
Vision blurred	0 (0.0%)	4 (2.3%)	1.000
Cognitive dysfunction	4 (57.1%)	27(15.4%)	0.017
**Personal history**			
Hypertension	5 (71.4%)	147 (84.0%)	0.325
Hyperhomocysteinemia	2 (28.6%)	54 (30.9%)	1.000
Diabetes mellitus	2 (28.6%)	53 (30.3%)	1.000
Coronary artery disease	1 (14.3%)	35 (20.0%)	1.000
Stroke	5 (71.4%)	146 (83.4%)	0.341
Smoking	4 (57.1%)	50 (28.6%)	0.198
Drinking	1 (14.3%)	31 (17.7%)	1.000
**Family history**			
Stroke	1 (14.3%)	25 (14.3%)	1.000
**White matter hyperintensity**			
PWMH (⩾2)	7 (100%)	175 (100%)	NA
DWMH (⩾2)	6 (85.7%)	165 (94.3%)	0.359
**LH**	6 (85.7%)	155 (88.6%)	0.583
**CMB**	3 (42.9%)	45 (25.7%)	0.382

Data represent the mean ± SD or the number (%) of subjects. Student's t-test for the continuous variables and Fisher's exact test for the dichotomized variables. $P<0.05$ was considered statistically significant.

Supplementary Table 3. Eight pathogenic variants identified in CSVD-related genes according to ACMG practice guidelines.

Gene & Location					ACMG	
Gene	Exon	SNP ID	Nucleotide	AminoAcid	InterVar	InterVar_evidence
NOTCH3	8	NA	c.1261C>T	p. Arg421Cys	US	PVS1=0 PS=[0, 0, 0, 0, 0] PM=[1, 1, 0, 0, 0, 0, 0] PP=[0, 0, 1, 0, 0, 0] BA1=0 BS=[0, 0, 0, 0, 0] BP=[0, 0, 0, 0, 0, 0, 0, 0]
NOTCH3	11	rs201118034	c.1630C>T	p. Arg544Cys	US	PVS1=0 PS=[0, 0, 0, 0, 0] PM=[0, 0, 0, 0, 0, 0, 0] PP=[0, 0, 1, 0, 0, 0] BA1=0 BS=[0, 0, 0, 0, 0] BP=[0, 0, 0, 0, 0, 0, 0, 0]
NOTCH3	11	rs764148985	c.1774C>T	p. Arg592Cys	US	PVS1=0 PS=[0, 0, 0, 0, 0] PM=[1, 1, 0, 0, 0, 0, 0] PP=[0, 0, 1, 0, 0, 0] BA1=0 BS=[0, 0, 0, 0, 0] BP=[0, 0, 0, 0, 0, 0, 0, 0]
NOTCH3	19	NA	c.3091C>T	p. Arg1031Cys	US	PVS1=0 PS=[0, 0, 0, 0, 0] PM=[1, 1, 0, 0, 0, 0, 0] PP=[0, 0, 1, 0, 0, 0] BA1=0 BS=[0, 0, 0, 0, 0] BP=[0, 0, 0, 0, 0, 0, 0, 0]
NOTCH3	23	NA	c.3784C>T	p. Arg1262Cys	US	PVS1=0 PS=[0, 0, 0, 0, 0] PM=[1, 1, 0, 0, 0, 0, 0] PP=[0, 0, 1, 0, 0, 0] BA1=0 BS=[0, 0, 0, 0, 0] BP=[0, 0, 0, 0, 0, 0, 0, 0]
HTRA1	8	rs147459330	c.1207C>T	p. Arg403Trp	US	PVS1=0 PS=[0, 0, 0, 0, 0] PM=[1, 1, 0, 0, 0, 0, 0] PP=[0, 0, 0, 0, 0, 0] BA1=0 BS=[0, 0, 0, 0, 0] BP=[0, 0, 0, 0, 0, 0, 0, 0]
HTRA1	8	rs751805574	c.1274+1G>A	NA	P	PVS1=1 PS=[0, 0, 0, 0, 0] PM=[0, 1, 0, 0, 0, 0, 0] PP=[0, 0, 1, 0, 0, 0] BA1=0 BS=[0, 0, 0, 0, 0] BP=[0, 0, 0, 0, 0, 0, 0, 0]
COL4A1	27	rs532972509	c.1937G>C	p. Gly421Ala	US	PVS1=0 PS=[0, 0, 0, 0, 0] PM=[0, 0, 0, 0, 0, 0, 0] PP=[0, 0, 1, 0, 0, 0] BA1=0 BS=[0, 0, 0, 0, 0] BP=[0, 0, 0, 0, 0, 0, 0, 0]

NA, Not Available; NF, Not Found. The amino acid is based on the corresponding cDNA position according to the reference sequences of *NOTCH3* (GRCh38, NM_000435), *HTRA1*(GRCh38, NM_002775), *COL4A1*(GRCh38, NM_001845).

P = Pathogenic, US = Uncertain significance;

Supplementary Figure 1. The amino acid sequence alignments show that p.R421C (*NOTCH3*), p.R544C (*NOTCH3*), p.R592C (*NOTCH3*), p.R1031C (*NOTCH3*), p.R1262C (*NOTCH3*), p.R403W (*HTRA1*), p.G646A (*COL4A1*) are highly conserved among the different species.

		421	544	592	1031	1262
HUMAN	NOTCH3	LCQCG**R**GYTGP	GTLCD**R**NVDDC	RSQPC**R**HGGKC	PGWSG**R**LCDIR	HGGQC**R**PSPGP
MOUSE	NOTCH3	LCQCG**R**GYTGP	GTLCE**R**NVDDC	RSQPC**R**YGGKC	PGWSG**R**LCDIQ	HGGQC**R**HSLGR
CANLF	NOTCH3	LCQCG**R**GYTGP	GTLCE**R**NVDDC	RSQPC**R**HGGKC	PGWSG**R**LCDLR	HGGQC**R**PSPGP
BOVIN	NOTCH3	LCQCG**R**GYTGP	GTVCE**R**NVDDC	RSQPC**R**HGGKC	PGWSG**R**LCDIR	HGGQC**R**PSPGP
PIG	NOTCH3	LCQCG**R**GYTGP	GTVCE**R**NVDDC	RSQPC**R**HGGKC	PGWSG**R**LCDIQ	HGGQC**R**PSPGP

		403			646
HUMAN	HTRA1	LKDRH**R**DFPDV	HUMAN	COL4A1	IVPLP**G**PPGAD
MOUSE	HTRA1	LKDRH**R**DFPDV	MOUSE	COL4A1	VVPLP**G**PPGAD
DANRE	HTRA1	LKGRL**R**DFPDI	BOVIN	COL4A1	VVPLP**G**PPGAD
BOVIN	HTRA1	LKDRH**R**DFPDV	HORSE	COL4A1	VVPLP**G**PPGAD
HORSE	HTRA1	LKDRH**R**DFPDV	CANLF	COL4A1	VVPLP**G**PPGAD
