# Supplementary figures and images for: Genetic Study of Cerebral Small Vessel Disease in Chinese Han Population
Source: Front Neurol. 2022 Mar 25;13:829438. doi: 10.3389/fneur.2022.829438 (PMC8990910; doi:10.3389/fneur.2022.829438)

Patient-1

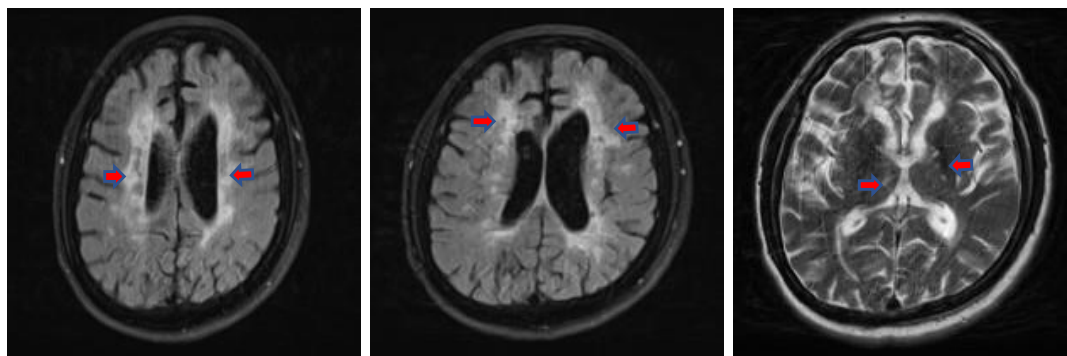

Patient-2

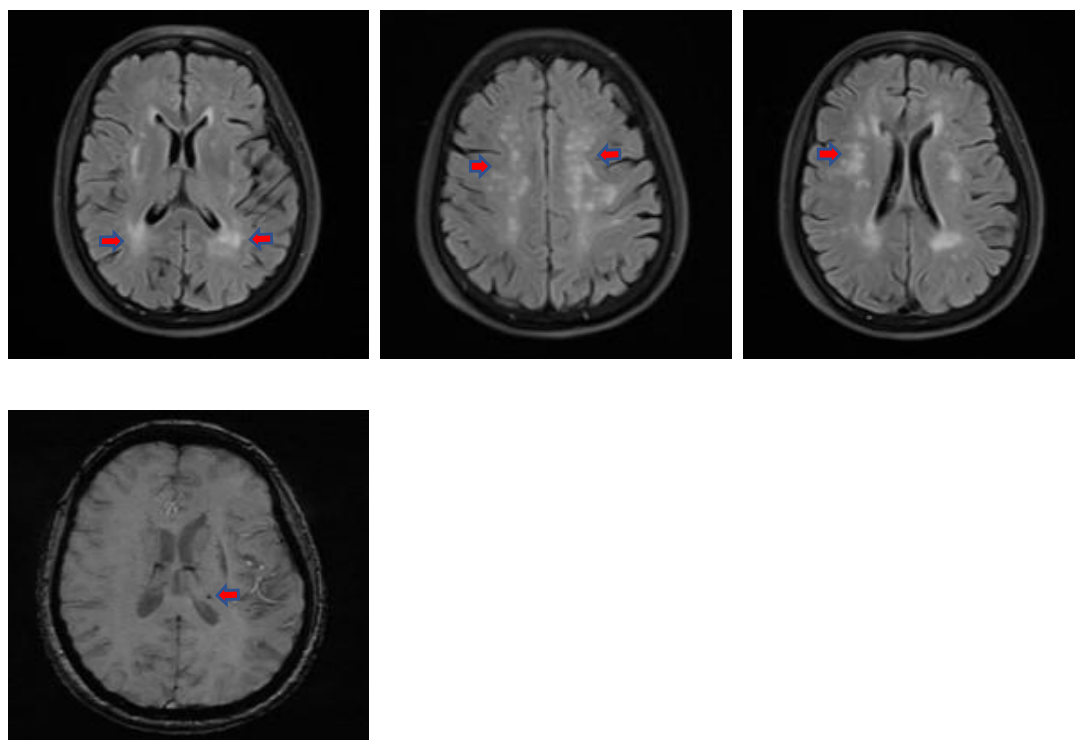

Patient-3

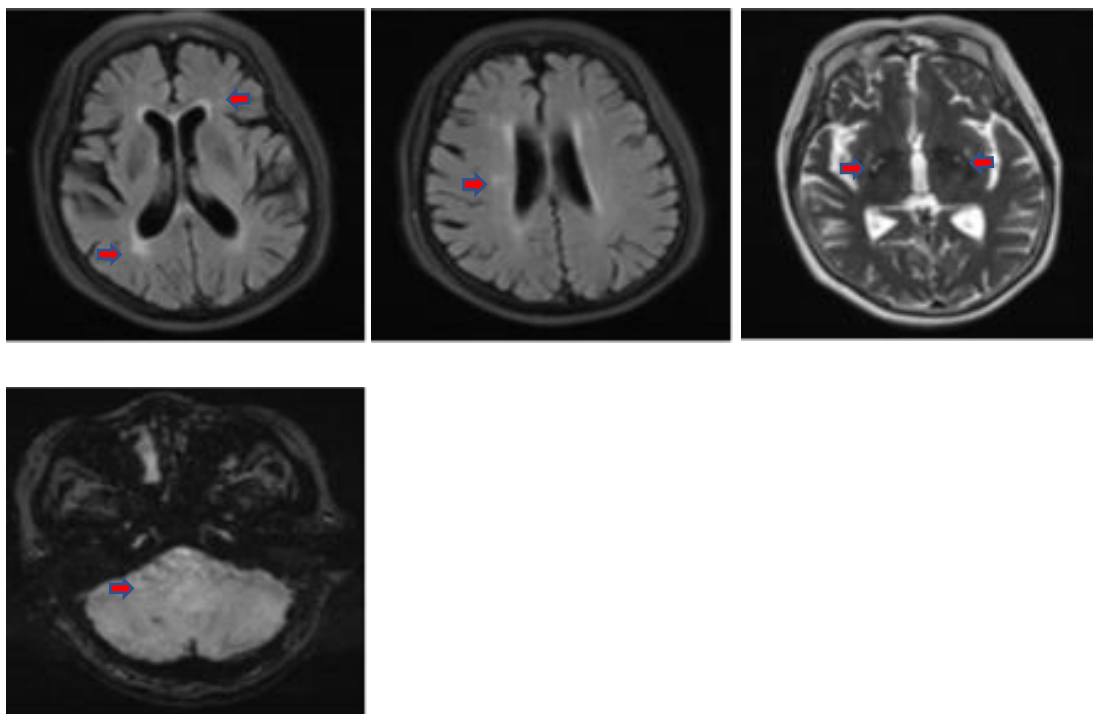

Patient-4

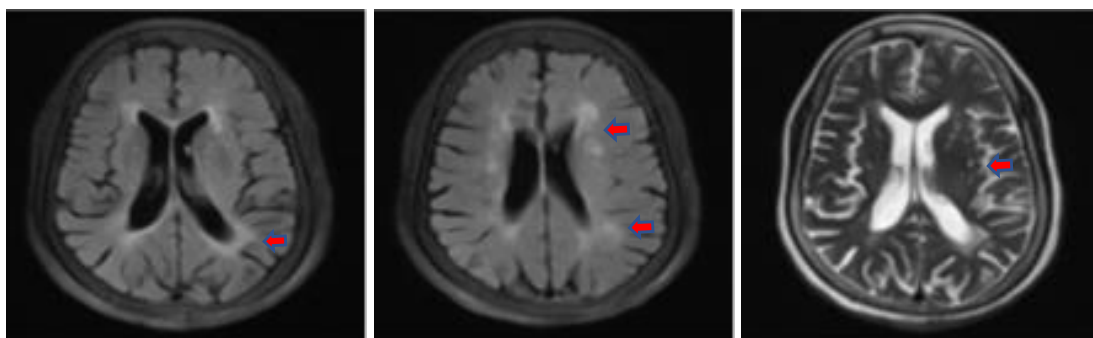

Patient-5

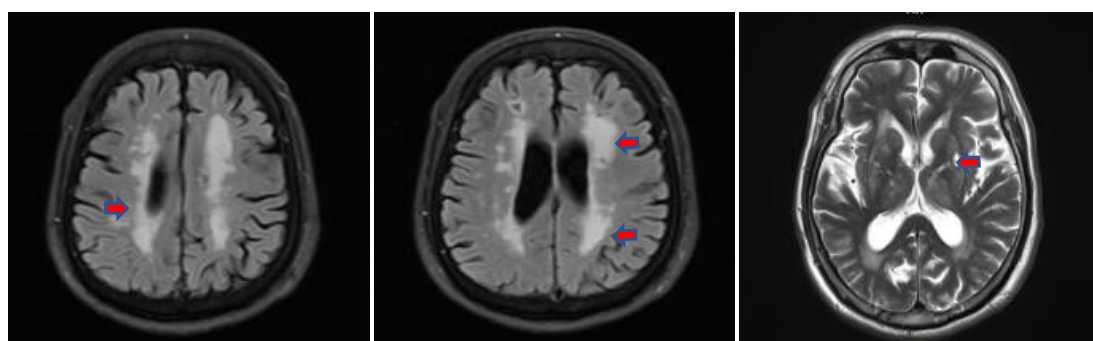

Patient-6

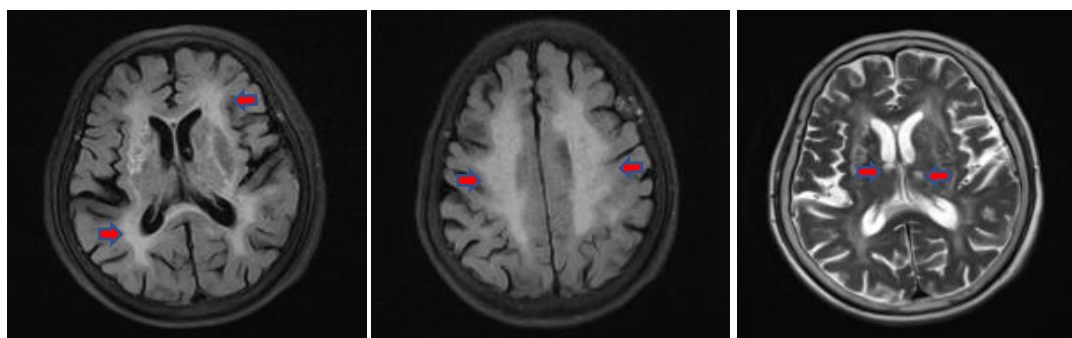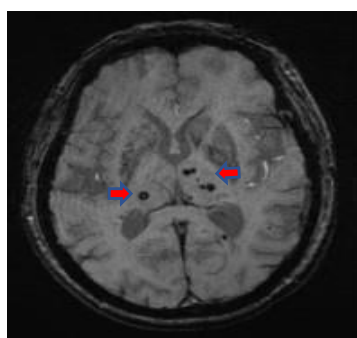

Patient-7

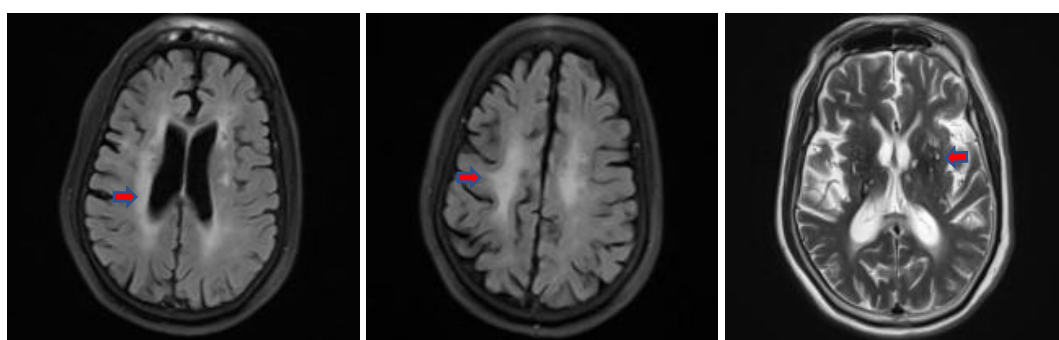

Supplement: Supplementary file 2 [file Data_Sheet_2.pdf]
